# Utilization and regional disparities of radiotherapy in cancer treatment in Japan: a longitudinal study using NDB open data

**DOI:** 10.1093/jrr/rrae100

**Published:** 2024-12-27

**Authors:** Kazuya Takeda, Rei Umezawa, Takaya Yamamoto, Noriyoshi Takahashi, Hiroshi Onishi, Keiichi Jingu

**Affiliations:** Department of Radiation Oncology, Tohoku University Graduate School of Medicine, 1-1 Seiryo-machi, Aoba-ku, Sendai, Miyagi 980-8574, Japan; Department of Radiation Oncology, South Miyagi Medical Center, 38-1 Nishi, Ogawara, Shibata, Miyagi 989-1253, Japan; Department of Radiation Oncology, Tohoku University Graduate School of Medicine, 1-1 Seiryo-machi, Aoba-ku, Sendai, Miyagi 980-8574, Japan; Department of Radiation Oncology, Tohoku University Graduate School of Medicine, 1-1 Seiryo-machi, Aoba-ku, Sendai, Miyagi 980-8574, Japan; Department of Radiation Oncology, Tohoku University Graduate School of Medicine, 1-1 Seiryo-machi, Aoba-ku, Sendai, Miyagi 980-8574, Japan; Department of Radiology, Faculty of Medicine, University of Yamanashi, 4-4-37 Takeda, Kofu, Yamanashi 400-8510, Japan; Department of Radiation Oncology, Tohoku University Graduate School of Medicine, 1-1 Seiryo-machi, Aoba-ku, Sendai, Miyagi 980-8574, Japan

**Keywords:** radiotherapy, IMRT, STI, NDB open data, regional disparity

## Abstract

The National Database of Health Insurance Claims and Specific Health Checkups of Japan (NDB) is a database that stores anonymized information on medical receipts and health checkups in Japan. The NDB Open Data is a publicly accessible summary table of the NDB database. To reveal annual trends and regional disparities in radiotherapy utilization in Japan, we analyzed the NDB Open Data tables for a 9-year period from 2014 to 2022. We extracted medical cost codes for radiotherapy management fees and specific types of radiotherapy, such as stereotactic irradiation (STI) and intensity-modulated radiotherapy (IMRT), to analyze nationwide changes over time. To investigate regional disparities, we counted the three subitems representing 3-dimensional conformal radiotherapy (3D-CRT), IMRT, and STI for each prefecture per year. The utilization of advanced radiotherapy techniques, such as IMRT (199% increase), increased, while the use of simpler forms of irradiation, such as 1 or 2-opposite fields irradiation (40% decrease), decreased in the period from 2014 to 2022. Regarding regional disparities, the coefficients of variation in 47 prefectures for 3D-CRT remained relatively stable at 0.17 in 2014 and 0.18 in 2022, while the coefficients of variation for IMRT and STI decreased from 0.64 and 0.39 in 2014 to 0.31 and 0.36 in 2022, respectively. The popularization of IMRT was correlated with the number of certified radiation oncologists in the prefecture. In conclusion, although the utilization of high-precision radiotherapy in Japan has been increasing and regional differences have been diminishing, there are still persistent disparities.

## INTRODUCTION

Radiation therapy has been advancing and spreading since its first clinical application for malignant tumors in 1896. In recent years, high-precision radiotherapy, such as intensity-modulated radiation therapy (IMRT) and stereotactic irradiation (STI), has been increasingly used for various types of cancers including prostate, head and neck, and lung cancers. However, the implementation of advanced irradiation techniques requires experienced staff and expensive equipment, which may limit the widespread use of advanced radiotherapy in actual treatment, causing discrepancies between urban and rural areas. Although there have been reports on the increasing use of IMRT and STI in Japan [1–3], the utilization rate remains low compared to the utilization rates in other developed countries. Nevertheless, there is a lack of research regarding regional disparities of high-precision radiotherapy in Japan.

The National Database of Health Insurance Claims and Specific Health Checkups of Japan (NDB) is an anonymized database that contains information on insurance claims and health checkups in Japan [4]. NDB Open Data is a publicly accessible and highly versatile dataset that provides fundamental tabulations of NDB data [5]. Previous studies have shown the usefulness of NDB Open Data for revealing the utilization of various treatment options and their changes in the Japanese healthcare system [6–8]. Furthermore, NDB Open Data includes data for each of Japan’s 47 prefectures. In Japan, with few exceptions, tertiary medical regions have been established for each prefecture, and there is a policy to provide advanced medical care within these regions. Therefore, the distribution of the number of radiotherapy claims per population in each prefecture can indicate the uniformity level in the utilization of radiotherapy throughout Japan. Consequently, we conducted an analysis of radiotherapy utilization data for each prefecture in NDB Open Data to elucidate the prevalence of high-precision radiotherapy in Japan and its regional disparities.

## METHODS

### Data acquisition

This study was conducted using public databases and does not constitute clinical research on human subjects. We obtained NDB Open Data tabulations for the fiscal years 2014 to 2022 from the official website of the Japanese Ministry of Health, Labour and Welfare [5]. In each year, we extracted the medical fee codes for radiation therapy management fees and special irradiation techniques shown in Table 1 and examined the annual changes in the number of claims. The radiotherapy management fee (classification code M000) can be billed for the creation of a dose distribution map and is billed once or twice per series of radiotherapy treatments, and this was used as a surrogate indicator for the number of radiotherapy treatments. For special irradiation techniques such as STI and particle therapy, radiation therapy administration fees (classification codes M001–2, M001–3 and M001–4) are billed in place of radiation therapy management fees, and these fees were used as a surrogate indicator for the number of cases. Additionally, the analysis included the classification code A400, which represents short-term inpatient treatment using the Gamma Knife. For the analysis of medical claims in this study, we used the term STI to refer to both single-fraction stereotactic radiosurgery and fractionated stereotactic radiotherapy. Based on the categorization of treatment items in Japanese insurance claims, the following classifications were used to identify each treatment type: Conventional 3-dimensional conformal radiotherapy (3D-CRT) was categorized as ‘Simple’ for one- or opposed two-field irradiation and ‘Complex’ for non-opposed two-field irradiation or three-field irradiation. Irradiation with four or more fields, moving irradiation, and conformation irradiation were classified as ‘Special.’ In this study, high-precision radiotherapy was defined as IMRT and STI. The claims were further categorized as ‘IMRT,’ ‘ STI with Gamma Knife’ and ‘STI with linac.’ The ‘Others’ category, which is billed for single-fraction radiotherapy, was also included in the analysis. The category of ‘Particle therapy’ included both proton beam therapy and carbon ion beam therapy. To investigate variations in claims across different regions, the total numbers of claims for 3D-CRT, IMRT and STI were aggregated by prefecture (Table 1). We used these selected treatment categories to examine the variations in utilization by prefecture. For regional analysis, prefectures were divided into six areas (North Japan, Kanto, Chubu, Kansai, Chugoku-Shikoku and Kyushu-Okinawa) based on the regional branches of the Japan Radiological Society, and claim numbers were aggregated accordingly. The Vital Statistics of Japan were used to reference for the population at each fiscal year for the entire country and each prefecture [9]. Information on the number of certified radiation oncologists in each prefecture was obtained from the Japanese Society of Radiation Oncology (JASTRO) for the available fiscal years (2012 and 2022).

**Table 1:** Evaluated medical fee codes

Medical fee code	Category name	Subcategory name	Medical action code
M000	Radiotherapy management fee	Simple	113 001 110, 180 019 010
		Complex	180 018 410, 180 019 110
		Special	180 018 510, 180 019 310, 180 019 210
		IMRT	180 031 710
M001–2	Stereotactic radiotherapy with gamma knife	STI with gamma knife	180 018 910
A400	Short stay surgery basic fee	Gamma knife in short stay	190 197 910, 190 198 010
M001–3	Radiotherapy with linac	STI with linac	180 019 710, 180 026 750
		Others	180 035 310
M001–4	Particle therapy	Proton therapy	180 055 110, 180 055 310, 180 046 710
		Heavy particle therapy	180 055 010, 180 055 210, 180 046 610

### Statistical analysis

Information on the number of claims for each treatment item was obtained and totals were calculated for each item. Annual changes were plotted on a line graph. To assess the extent of change, the percent change was calculated using the values from 2014 to 2022. The number of claims for each year was also calculated for each prefecture for the three selected claim items mentioned above. In NDB Open Data, values for prefectures with less than 10 claims per year for each medical action code are masked to protect personal information. In cases in which prefectures had missing values, estimated values calculated from the population of each prefecture were filled in. Data for IMRT in 2015 and 2017 were excluded because the number of claims could not be obtained for all prefectures. Based on the number of claims and the population in that year, we calculated the number of claims per 100 000 population.

To compare different treatments, the relative number of claims per 100 000 population in each prefecture was calculated with the number of claims per 100 000 population in Japan used as the base unit. The relative value of the number of claims per prefecture was visualized by plotting it on a map. For quantitative evaluation, annual changes were plotted using the coefficient of variation of the values. Correlation coefficients were used to assess the correlation between the number of certified radiation oncologists per prefecture and the number of claims for various treatment modalities. Since data for the relevant years were not available, the analysis was performed by fitting the nearest available data for the number of certified radiation oncologists from 2012 and 2022 to the survey years of 2014 and 2022, respectively. To visualize the variation and temporal changes in the utilization of each treatment modality across regions, the annual number of claims in each region was plotted for each treatment category.

All statistical analyses were performed using JMP Pro 17.1.0 (JMP Statistical Discovery LLC, NC).

## RESULTS

### Nationwide number of radiation therapy claims

**Fig. 1:**
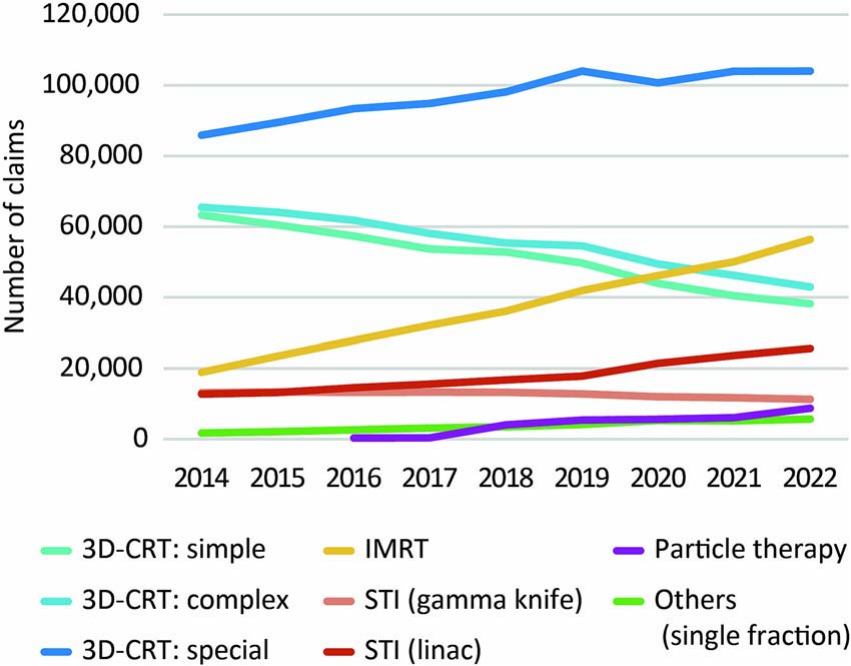
Temporal changes in number of medical fee claims for various types of radiotherapy.

Figure 1 shows the annual number of claims for radiotherapy nationwide on an annual basis. The change in the number of claims for each type of treatment in 2022 from the base year of 2014 is shown below. Claims for ‘Simple’ and ‘Complex’ 3D-CRT declined to 60.4% and 65.6%, respectively, while claims for ‘Special,’ which mainly includes four or more multi-field irradiations, increased to 121.1%. As for high-precision radiotherapy, IMRT showed a large increase to 298.6%. STI using a linear accelerator increased to 201.6%, while STI using the Gamma Knife decreased slightly to 86.0%. The ‘Others’ category of M001–3, which applies to single irradiation for palliative purposes, increased to 326.1%. Particle therapy was covered by insurance for some diseases in 2018, which led to an increase in the number of claims since then.

### Number of medical fee claims per prefecture

**Fig. 2:**
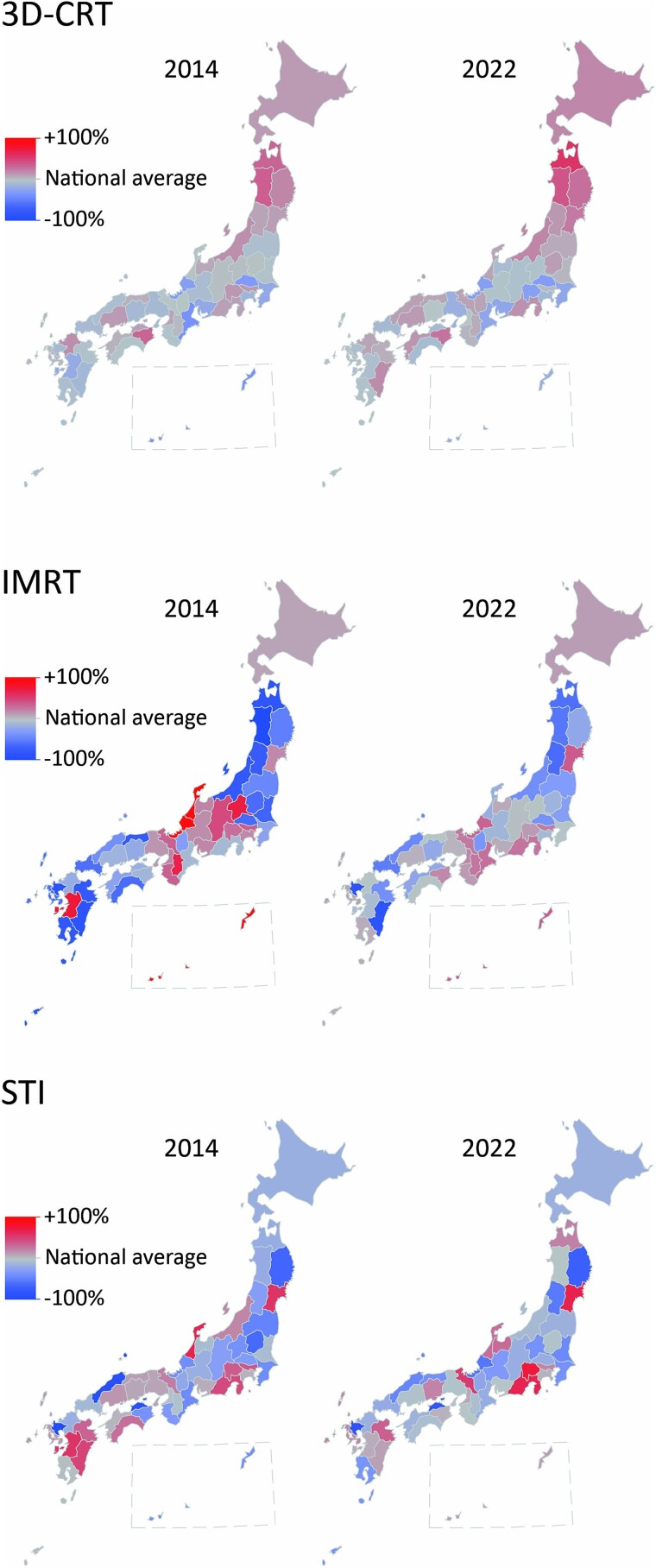
Map plot of the number of claims per 100 000 population in each prefecture relative to the national average each year for three different types of radiotherapy.

**Fig. 3:**
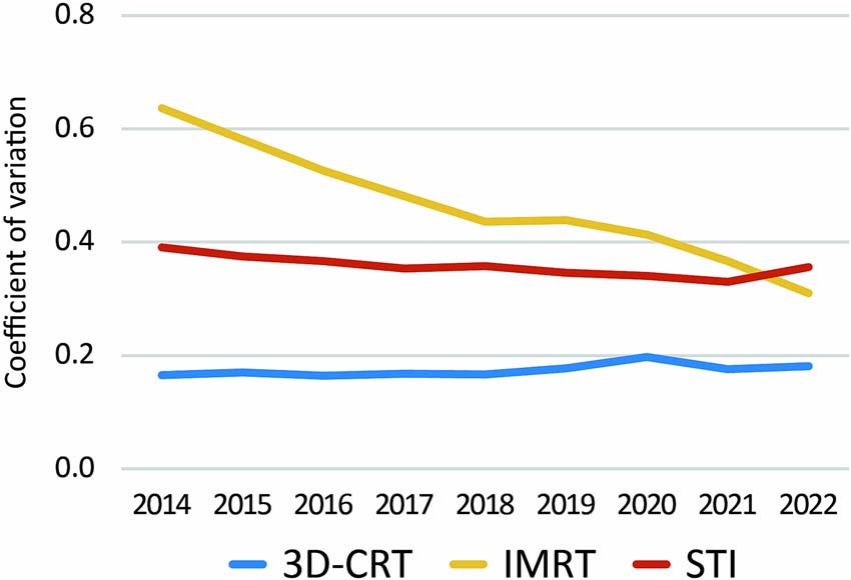
Temporal changes in coefficient of variation of count of three different types of radiotherapy.

**Fig. 4:**
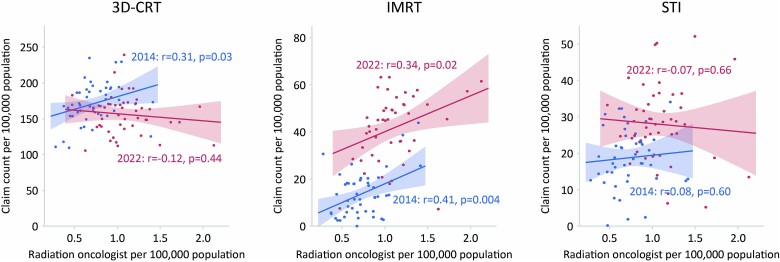
Distribution of the number of certified radiation oncologists per 100 000 population and number of claims per treatment in each prefecture in 2014 and 2022. Data for the number of certified radiation oncologists were obtained from the nearest available year, 2012 and 2022 for 2014 and 2022, respectively.

**Fig. 5:**
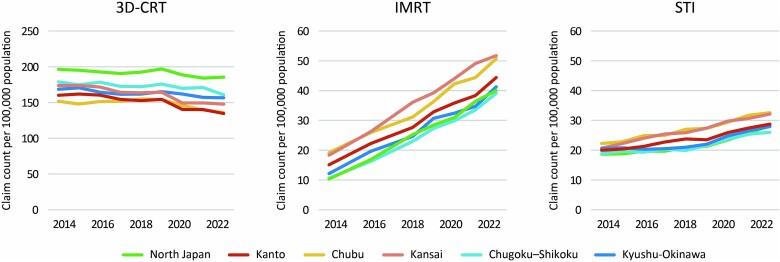
Changes over time in the use of three different types of radiotherapy per population across each region.

A summary of the three different types of radiotherapy is presented in Table 2. Due to missing data, the values for IMRT in 2015 and 2017 were excluded. After excluding these values, missing data were found in 0.1–0.7% of cases, for which estimates were substituted. The distributions of the numbers of medical fee claims per prefecture in 2014 and 2022 for the three treatment types are shown in Fig. 2. The map plot reveals that the distribution bias towards the central part of the country observed in 2014 for IMRT was reduced in 2022. For quantitative evaluation, a plot of the year-to-year changes in the coefficients of variation between prefectures is shown in Fig. 3. The coefficients of variation for 3D-CRT remained relatively stable at 0.16 in 2014 and 0.18 in 2022. In contrast, the coefficient of variation for IMRT decreased from 0.64 in 2014 to 0.31 in 2022, approaching the coefficient of variation for 3D-CRT. For STI, the coefficient of variation decreased from 0.39 in 2014 to 0.33 in 2020 but slightly increased to 0.36 in 2022. Figure 4 shows the correlation between the number of certified radiation oncologists per 100 000 population and the number of claims for various treatment modalities per prefecture. In 3D-CRT, data in 2014 showed a positive correlation, but no significant correlation was observed in 2022 (2014: r = 0.31, *P* = 0.03; 2022: r = −0.12, *P* = 0.44). IMRT showed a positive correlation in both survey years (2014: r = 0.41, *P* = 0.004; 2022: r = 0.34, *P* = 0.02) and STI did not show a significant correlation in both years (2014: r = 0.08, *P* = 0.60; 2022: r = −0.07, *P* = 0.66). Figure 5 shows the changes over time in the use of three different types of radiotherapy per population across regions. Throughout the study period, advanced treatment techniques such as IMRT and STI were consistently utilized more frequently in the Kanto, Chubu and Kansai regions.

**Table 2:** Detail of accumulated 3 treatment types

Treatment type	Medical action code	Total record (2014–2022)	Masked data (claim number based)
3D-CRT	113 001 110, 180 019 010, 180 018 410, 180 019 110, 180 018 510, 180 019 310, 180 019 210	1 832 472	0.6%
IMRT	180 031 710	220891^a^	0.1%^a^
STI	180 018 910, 190 197 910, 190 198 010, 180 019 710, 180 026 750	275 464	0.7%

^a^Due to missing data, IMRT was tabulated for 7 years of data, excluding 2015 and 2017 values.

## DISCUSSION

In this study, we analyzed data obtained from NDB Open Data for a nine-year period from 2014 to 2022 in order to examine the prevalence of high-precision radiotherapy in Japan and the regional disparities associated with it. Our nationwide analysis of claim records revealed a notable increase in claims for high-precision radiotherapy such as IMRT and STI. Moreover, analysis of the data at the prefecture and regional level revealed persisting disparities in the number of claims for high-precision radiotherapy across different areas although the extents of disparities were decreasing. To the best of our knowledge, this study is the first study to investigate regional disparities in radiotherapy utilization using NDB Open Data.

NDB Open Data, which is a publicly available dataset derived from the NDB that includes data for health insurance claims data in Japan, was used in this study. There are comparable administrative claims databases in other countries such as CMS Medicine Data in the United States, the Clinical Practice Research Datalink in the United Kingdom, and the National Health Insurance Service in South Korea. Japan and South Korea have universal health insurance systems with most of the population being covered by health insurance, and the database coverage is extremely high. When conducting epidemiological studies utilizing administrative claims databases, it is crucial to address concerns regarding potential biases arising from variations in the characteristics of the enrollees in each health insurance plan. In this respect, the NDB is well-suited for conducting comparative investigations into the utilization of radiotherapy across different regions, free from such biases.

Analysis of the number of claims for radiotherapy nationwide revealed a decline in simpler radiotherapy techniques, while the number of claims for more advanced treatment techniques such as multi-field irradiation, IMRT, and STI increased. Although this study focused on insurance claims and a direct comparison of the actual numbers of patients treated was not possible, the results indicate that there was an increase in the utilization of high-precision radiotherapy in Japan. Regarding STI, the use of linear accelerators for STI increased by 102% during the 9-year period, while the use of Gamma Knife for STI decreased by 14%. This trend may be attributed to the expanded insurance reimbursement for STI in trunk tumors, the enhanced performance of linear accelerators that enable STI without the need for a dedicated STI machine, and the widespread adoption of robotic arm linear accelerators. Recent clinical outcomes have demonstrated the superiority of high-precision radiotherapy such as IMRT and STI. IMRT has been reported to reduce adverse events in several types of cancer including head and neck cancer, anal cancer, and prostate cancer [10–12]. In prostate cancer treatment, IMRT enabled dose escalation, leading to superior disease control compared with a historical control [13]. Likewise, high-dose irradiation with STI has been shown to result in better outcomes than the outcomes of conventional radiotherapy for early-stage lung cancer [14]. Consequently, the demand for high-precision radiotherapy is increasing and is expected to continue increasing.

While the absolute number remains small, the number of claims filed under ‘Others’ for single radiation therapy for palliative purposes more than tripled from 2014 to 2022. In Japan, fractionated irradiation schemes such as 30 Gy in 10 fractions and 20 Gy in 5 fractions have been widely used in palliative radiotherapy [15]. However, there is a rising trend in the use of single-fraction irradiation based on evidence showing the efficacy of single-fraction irradiation, such as 8 Gy in 1 fraction, in palliation of bone metastasis pain [16, 17]. These observations indicate a preference for treatment methods with shorter durations for both curative treatment and palliative treatment. In Japan, the JASTRO conducted a nationwide survey and established a national registry of radiotherapy facilities, which also revealed an increase in the utilization of high-precision radiation therapy and a tendency towards shorter treatment durations [1]. These findings are consistent with the results of the NDB Open Data analysis in this study.

Recently, studies conducted in the United States and Canada have indicated a correlation between limited access to radiation therapy and unfavorable outcomes in cancer treatment [18, 19]. In this study, we analyzed the availability of radiotherapy across different regions by comparing the number of claims per prefecture. In particular, a large decrease in the coefficient of variation for the use of IMRT across prefectures was observed between 2014 and 2022. In Japan, policies have been implemented since the enactment of the Cancer Control Act in 2006 to promote equal access to cancer treatment and improve services in rural areas. These measures partly enforced adequate treatment facilities and personnel for high-precision radiotherapy in rural regions. However, in 2022, the coefficient of variation for the number of claims among prefectures remained relatively high for IMRT and STI compared to that for 3D-CRT. In South Korea, it has been reported that although the utilization of IMRT and STI is increasing, there is a disparity in usage rates between capital and non-capital areas [20]. These results highlight the need for further measures to achieve true equality in access to these treatments in countries where high-precision radiotherapy is in the process of spreading.

In our analysis of the number of certified radiation oncologists per prefecture and the numbers of medical fee claims for different treatment techniques, a significant correlation was found between the number of certified radiation oncologists and the number of claims for IMRT in both 2014 and 2022. Unlike 3D-CRT and STI, insurance claims for IMRT in Japan require at least two radiation oncologists at a single facility in principle. This requirement may have limited the use of IMRT in areas with a limited number of radiation oncologists. A recent study in Japan showed that the number of treatment planning systems per radiotherapy device and the number of medical physicists per million population were correlated with and the rate of high-precision radiotherapy implementation [3]. Consequently, to promote the use of IMRT in Japan, it is necessary to increase the staff for radiotherapy including certified radiation oncologists. Also, it is desirable to relax the requirements for staff numbers and enable radiotherapy facilities with one radiation oncologist to perform IMRT using supporting frameworks, such as radiation planning assistant, remote planning system and artificial intelligence.

The regional analysis showed that advanced radiotherapy techniques, such as IMRT and STI, were used more frequently in the Kanto, Chubu and Kansai regions, which include Japan’s three major metropolitan areas: Tokyo, Osaka and Nagoya. Although the overall coefficients of variation in the use of IMRT and STI decreased over time, absolute differences between regions persisted. On the other hand, 3D-CRT was used more frequently in non-central regions such as North Japan and the Chugoku-Shikoku area, suggesting the potential disparity in access to advanced radiotherapy techniques in rural areas.

This study has several limitations. Firstly, although NDB Open Data is based on insurance claims and provides a high population coverage, it does not provide information on the actual number of patients treated. Consequently, the actual number of patients receiving radiotherapy and the proportion of patients receiving each treatment option could not be accurately determined in this study. To obtain the precise number of patients treated, a detailed investigation utilizing JASTRO’s nationwide survey or NDB’s individual data is necessary. Secondly, NDB Open Data masks data for items with 10 or fewer cases to safeguard personal information. We imputed the missing values based on the population of each region, but this approach may have led to a slight underestimation of regional disparities. Thirdly, the unit of analysis used in this study was prefectures. Prefectures in Japan vary significantly in size, ranging from a minimum of 1877 km^2^ to a maximum of 83 424 km^2^, and the distance to the nearest radiotherapy facility differs greatly across regions. This problem of accessibility was not considered in this study. Fourthly, it was not possible to analyze the data at the level of smaller medical care regions. Japanese policy requires that advanced medical care is provided in tertiary medical regions while routine cancer treatment is provided in smaller secondary medical regions (335 regions in the country in 2021). Analyzing data at this level would likely reveal even larger disparities. However, due to data masking, we could not obtain information for secondary medical regions, and analysis was conducted using tertiary medical regions as the unit of analysis.

In conclusion, this study has elucidated the prevalence of high-precision radiotherapy in Japan and its regional disparities. Although the utilization of high-precision radiotherapy is increasing in Japan and regional disparities are diminishing, disparities still persist, necessitating further efforts to achieve equality in the use of high-precision radiotherapy.

## PRESENTATION AT A CONFERENCE

The 62nd Annual Meeting of Japan Society of Clinical Oncology.
